# Photocatalytic and anticancer potentials of green-synthesized neodymium-doped zinc oxide nanoparticles

**DOI:** 10.1007/s13205-026-04932-7

**Published:** 2026-07-06

**Authors:** L. Umaralikhan, M. Ashokkumar, K. J. Senthil Kumar

**Affiliations:** 1 Department of Physics, Jamal Mohamed College (Autonomous), Affiliated to Bharathidasan University, Tiruchirappalli, Tamil Nadu 620020 India; 2 Department of Physics, Saveetha School of Engineering, Saveetha Institute of Medical and Technical Sciences (SIMATS), Saveetha University, Chennai, Tamil Nadu 602 105 India; 3 Center for General Education, National Chung Hsing University, Taichung, 402 Taiwan

**Keywords:** ZnO nanoparticles, Rare-earth element, Neodymium doping, Photocatalytic activity, Cytotoxicity green synthesis

## Abstract

Zinc oxide nanoparticles (ZnO NPs) doped with rare-earth elements such as neodymium (Nd) demonstrate improved optical, catalytic, and biomedical functionalities, making them promising for multifunctional applications. However, investigations into these properties in green-synthesized Nd-doped ZnO NPs are still limited. Thus, this study aimed to synthesize, characterize, and evaluate the environmental and biomedical potentials of pure and Nd-doped ZnO NPs using *Psidium guajava* (*P. guajava*) leaf extract (PGLE). Pure and Nd-doped ZnO nanoparticles were synthesized using *PGLE*. Their structural, morphological, and optical properties were characterized by X-ray diffraction (XRD), high-resolution transmission electron microscopy (HRTEM), Raman spectroscopy, photoluminescence (PL), and X-ray photoelectron spectroscopy (XPS). Photocatalytic performance was assessed through methylene blue degradation under sunlight, while cytotoxicity and apoptosis were evaluated in MCF-7 human breast cancer cells using acridine orange/ethidium bromide (AO/EB) staining. XRD and HRTEM analyses confirmed the hexagonal wurtzite structure of ZnO and revealed a notable reduction in particle size with Nd doping. Raman spectra and PL results indicated that Nd incorporation enhanced defect states and influenced luminescence properties. XPS verified successful Nd substitution, introducing lattice strain and defect sites. Photocatalytic studies showed ~ 99% methylene blue degradation within 150 min, with strong reusability. Cytotoxic assays revealed lower cell viability for Nd-ZnO (22%) compared to pure ZnO (24%), with AO/EB staining confirming apoptosis as the primary mode of cell death. Nd doping into ZnO lattices enhances reactive oxygen species (ROS) generation, improving photocatalytic efficiency and anticancer activity. The green synthesis using *P. guajava* leaf extract demonstrates a sustainable route for developing multifunctional Nd-ZnO-based nanomaterials for environmental and biomedical applications.

## Introduction

Zinc oxide (ZnO) is a multifunctional semiconductor material widely recognized for its exceptional optical, electrical, and photocatalytic properties. Belonging to the II–VI semiconductor family, ZnO exhibits a direct wide bandgap of approximately 3.37 eV and a high exciton binding energy of 60 meV at room temperature. These intrinsic characteristics render ZnO highly versatile for applications in photocatalysis, optoelectronics, piezoelectric devices, sensors, cosmetics, and biomedical systems (Borysiewicz 2019). At the nanoscale, ZnO nanoparticles (ZnO NPs) demonstrate pronounced quantum confinement, enhanced surface-to-volume ratios, and superior reactivity, which amplify their performance in energy conversion, pollutant degradation, antimicrobial treatments, and drug delivery applications (Dey 2025). The photocatalytic efficiency of ZnO stems from its capacity to generate electron–hole pairs upon exposure to ultraviolet (UV) or solar irradiation. The excited electrons and holes initiate redox reactions that produce reactive oxygen species (ROS), including hydroxyl radicals (•OH), superoxide anions (O₂•⁻), and hydrogen peroxide (H₂O₂), which can oxidize organic pollutants and inactivate pathogens. However, pristine ZnO faces significant limitations such as rapid charge recombination, photocorrosion, and poor visible-light absorption due to its wide bandgap (Aina et al. 2025). To address these challenges, strategies such as metal and non-metal doping, surface modification, and heterojunction coupling have been explored to improve light absorption, prolong carrier lifetimes, and enhance photocatalytic stability.

Among various doping strategies, **rare-earth element doping** has emerged as a particularly effective approach to modulate the electronic, structural, and optical properties of ZnO. Rare-earth elements such as neodymium (Nd), cerium (Ce), samarium (Sm), lanthanum (La), and gadolinium (Gd) possess partially filled 4f orbitals that introduce localized states within the ZnO bandgap (Pratomo et al. 2025). These energy states act as charge-trapping centers, reducing electron–hole recombination and extending charge carrier lifetimes. Furthermore, the incorporation of rare-earth ions alters lattice strain, induces oxygen vacancies, and modifies defect chemistry, thereby enhancing surface activity and visible-light responsiveness (Sood et al. 2025). Consequently, rare-earth-doped ZnO NPs often display improved photocatalytic performance, increased chemical stability, and stronger luminescence compared to undoped ZnO. Among rare-earth dopants, **neodymium (Nd**^**3**^**⁺)** is particularly attractive due to its efficient 4f–4f transitions, suitable ionic radius (close to Zn2⁺), and high optical activity. Substituting Zn^2^⁺ with Nd3⁺ slightly distorts the ZnO lattice and creates oxygen vacancies, enhancing visible-light absorption and improving photocatalytic activity under solar illumination (Tarasenka et al. 2022). Several studies have reported that Nd-doped ZnO exhibits significantly enhanced degradation efficiency toward dyes such as methylene blue (MB), and rhodamine 6G, as well as excellent reusability and chemical stability (Pascariu et al. 2023; Satpal and Athawale 2018). Moreover, Nd incorporation can decrease crystallite size and increase surface area, offering more active sites for photocatalytic reactions (Divya et al. 2025). Beyond photocatalysis, Nd3⁺ doping also improves ZnO’s optical and magnetic behavior, expanding its potential in optoelectronic and biomedical fields (Wu et al. 2023).

In the biomedical domain, ZnO NPs have gained increasing attention for their antimicrobial, antioxidant, anticancer, and wound-healing properties (Dey 2025; Mohammed et al. 2025; Xiao et al. 2025). The antimicrobial efficacy of ZnO arises from the combined effects of ROS generation, Zn2⁺ ion release, and direct membrane disruption, leading to bacterial inactivation. When doped with rare-earth elements, these nanoparticles exhibit improved oxidative stress generation and enhanced antibacterial potency against multidrug-resistant pathogens (Mohammed et al. 2025). Furthermore, ZnO NPs are inherently biocompatible and have demonstrated potential in tissue engineering, bone regeneration, and drug delivery systems due to their controlled ion dissolution and bioactive surface properties (Dey 2025). Nd3⁺ doping adds functional benefits, such as luminescence and imaging capability, making Nd-doped ZnO a promising candidate for bioimaging and photodynamic therapy (Carofiglio et al. 2020). These multifunctional attributes underscore the growing significance of rare-earth-doped ZnO NPs as next-generation materials bridging environmental remediation and biomedical applications.

Parallel to advancements in material modification, **the green synthesis of nanoparticles** has emerged as a sustainable and environmentally benign alternative to conventional physical and chemical synthesis methods. Traditional synthesis often involves high temperatures, toxic solvents, and hazardous reducing agents, leading to environmental concerns and limited biomedical compatibility (Gupta et al. 2023). In contrast, green synthesis leverages biological systems, including plants, microorganisms, or biopolymers as reducing and stabilizing agents under mild, eco-friendly conditions. Among these, **plant-mediated synthesis** has become particularly attractive due to the abundance of phytochemicals such as polyphenols, flavonoids, terpenoids, alkaloids, and sugars, which serve dual functions: reducing metal ions to nanoscale counterparts and stabilizing the resulting nanoparticles through capping interactions (Gupta et al. 2023).

Several plant systems have been explored for green synthesis, *Psidium guajava* **(guava)** (*P. guajava*) stands out as a promising bioresource owing to its rich phytochemical profile and extensive ethnomedicinal relevance (Huynh et al. 2025; Kumar et al. 2021). Widely distributed across tropical and subtropical regions, *P. guajava* has been used traditionally to treat gastrointestinal disorders, respiratory infections, wounds, and fever (Kumar et al. 2021). Its leaves, fruits, and bark contain diverse bioactive compounds, such as flavonoids (quercetin, kaempferol), phenolic acids (gallic and ferulic acids), tannins, saponins, terpenoids, and ascorbic acid which are exhibited strong antioxidant, anti-inflammatory, antidiabetic, and antimicrobial properties (Kumar et al. 2021). These phytoconstituents are particularly effective in reducing metal precursors and stabilizing nanoparticles during green synthesis. In addition, utilization of *P. guajava* extract in nanoparticle synthesis has been reported for various metals and metal oxides, including silver, gold, and zinc oxide (Patil and Rane 2020). These studies demonstrated controlled particle morphologies, narrow size distributions, and enhanced surface reactivity. Furthermore, guava-derived nanoparticles exhibit remarkable biological and catalytic performances, attributed to the synergistic effects between the phytochemical capping layer and the intrinsic properties of the nanomaterial (Patil and Rane 2020).

Although numerous studies have explored the synthesis and applications of ZnO nanoparticles, most conventional methods rely on chemical or physical routes that involve toxic reagents, high energy input, and limited biocompatibility. While green synthesis using plant extracts has gained attention, only a few studies have systematically examined the influence of *rare-earth metal doping* particularly, neodymium on the structural, optical, and biomedical performance of biosynthesized ZnO nanoparticles. Furthermore, previous studies often focus on either photocatalytic or biological activities, but rarely integrates both aspects within a single biogenic nanomaterial system. The synergistic relationship between Nd-induced defect modulation, ROS generation, and apoptosis-mediated anticancer mechanisms remains underexplored. Moreover, *P. guajava*, despite its rich phytochemical profile and proven medicinal properties, has been scarcely utilized as a bio-reductant and stabilizing agent for synthesizing rare-earth-doped ZnO NPs. However, comprehensive investigations linking *P. guajava*-mediated green synthesis with the effects of Nd-doping on the multifunctional properties of ZnO NPs, including optical, photocatalytic, and anticancer activities remain limited. Therefore, the present study aimed to design, synthesize, and characterize both pure and Nd-doped ZnO NPs using *P. guajava* leaf extract as a natural reducing and stabilizing agent. The synthesized nanoparticles were systematically evaluated for their structural, morphological, and optical characteristics, dopant incorporation, photoluminescence behavior, photocatalytic degradation efficiency, and anticancer potential against human breast cancer (MCF-7) cells.

## Materials and methods

### Plant material and leaf extract preparation

Fresh, healthy leaves of *Psidium guajava (P. guajava)* were collected from local cultivars located in Tiruchirappalli District, Tamil Nadu, India (10.7905° N, 78.7047° E). The plant material was taxonomically identified and authenticated by Dr. A. Aslam, Associate Professor of Botany and Fellow of the Indian Association for Angiosperm Taxonomy (FIAAT), Department of Botany, Jamal Mohamed College (Autonomous), Affiliated to Bharathidasan University, Tiruchirappalli—620 020, Tamil Nadu, India. A voucher specimen was prepared and deposited in the departmental herbarium under the accession number JMCH:2025–26/02 for future reference. The collected leaves were washed thoroughly under running tap water to remove adhering dust and other contaminants, followed by rinsing with double-distilled water. The cleaned leaves were air-dried at ambient temperature under shade to prevent the degradation of heat-sensitive phytochemicals. Subsequently, 10 g of finely chopped fresh leaves were mixed with 100 mL of double-distilled water and heated at 80 °C for 20 min under constant stirring to facilitate the extraction of bioactive compounds rich extract (PGLE). The mixture was cooled to room temperature and filtered through Whatman No. 1 filter paper to obtain a clear aqueous extract. The filtrate was collected in an airtight amber glass container and stored at 4 °C until further use for the green synthesis of nanoparticles.

### Preparation of ZnO and Nd-doped ZnO nanoparticles using P. guajava leaf extract

Zinc oxide (ZnO) nanoparticles were synthesized via a green route using the aqueous leaf extract of *P. guajava* (PGLE) as a bioreducing and stabilizing agent. A 0.1 M aqueous solution of zinc nitrate hexahydrate [Zn(NO₃)₂·6H₂O] was prepared and mixed with 100 mL of freshly prepared PGLE under continuous magnetic stirring at 50 °C for 35 min to facilitate reduction and nucleation. The obtained colloidal suspension was subsequently dried at 60–70 °C to yield a solid precursor, which was then subjected to calcination at 600 °C for 4 h in a muffle furnace to achieve phase-pure ZnO nanoparticles. For the synthesis of neodymium-doped ZnO (Nd–ZnO) nanoparticles, a 0.005 M solution of neodymium nitrate hexahydrate [Nd(NO₃)₃·6H₂O] was mixed with a 0.095 M solution of Zn(NO₃)₂·6H₂O to maintain a total metal ion concentration of 0.1 M. The mixture was processed under identical conditions as the pure ZnO synthesis (Nd-ZnO- NPs). The resulting powders were collected, finely ground using an agate mortar, and stored in airtight containers for subsequent physicochemical and biological characterizations.

### Characterization techniques

The crystalline structure of the synthesized nanoparticles (NPs) was analyzed using an X’PERT PRO PANalytical X-ray diffractometer with Cu Kα radiation (λ = 1.5406 Å) operating at 40 kV and 30 mA. Further structural and morphological insights were obtained by High-Resolution Transmission Electron Microscopy (HRTEM, JEOL JEM-2100F) operated at 200 kV. The Selected Area Electron Diffraction (SAED) patterns were recorded to assess the crystallinity and phase purity. The elemental composition and distribution were investigated through energy-dispersive X-ray analysis (EDAX) and X-ray photoelectron spectroscopy (XPS). The FT-IR spectra were recorded on a Perkin-Elmer spectrometer over the 400–4000 cm⁻1 range at a spectral resolution of 4 cm⁻1, using the KBr pellet method. The optical absorption characteristics of the nanoparticles were studied using a Lambda 35 UV–Visible spectrophotometer within the 300–700 nm wavelength range, employing quartz cuvettes with a 1 cm optical path length.

### Photocatalytic activity

The photocatalytic efficiency of the synthesized pure and neodymium-doped ZnO nanoparticles (Nd–ZnO NPs) was evaluated through the degradation of methylene blue (MB) dye under natural sunlight irradiation. A 250 mL aqueous MB solution (10 ppm) was prepared and supplemented with the catalyst at a concentration of 500 mg/L. Prior to illumination, the suspension was magnetically stirred in the dark for 60 min to establish adsorption–desorption equilibrium between dye molecules and the catalyst surface.

Following equilibration, the reaction mixture was exposed to sunlight for 150 min, and aliquots were collected at 30 min intervals to monitor the degradation process. The residual dye concentration was quantified spectrophotometrically by measuring the absorbance at 664 nm, the characteristic wavelength of MB. The photocatalytic degradation efficiency (D%) was calculated using Eq. (1):

1 $$\mathrm{D} \mathrm{\%} =\frac{{C}_{0} - {C}_{t}}{{C}_{0} } \times 100\mathrm{\%}$$

where *C₀* and *Cₜ* denote the initial and time-dependent concentrations of MB, respectively.

For recyclability studies, the used nanoparticles were recovered by centrifugation, thoroughly rinsed with deionized water, and dried in a vacuum oven at ambient temperature for 8 h. The regenerated catalysts were subsequently reused under identical experimental conditions to evaluate their photocatalytic stability and reusability.

### In vitro* cytotoxicity and apoptosis analysis*

The cytotoxic potential of pure and neodymium-doped ZnO nanoparticles (Nd–ZnO NPs) was evaluated against the human breast adenocarcinoma cell line (MCF-7) using the 3-(4,5-dimethylthiazol-2-yl)-2,5-diphenyltetrazolium bromide (MTT) assay, followed by acridine orange/ethidium bromide (AO/EB) staining to assess apoptosis and necrosis.

#### Cell culture and treatment

MCF-7 cells were obtained from the National Centre for Cell Science (NCCS, Pune, India) and cultured in Dulbecco’s Modified Eagle Medium (DMEM) supplemented with 10% fetal bovine serum (FBS), 100 U/mL penicillin, and 100 μg/mL streptomycin. The cells were maintained in a humidified incubator at 37 °C with 5% CO₂. For cytotoxicity testing, cells were seeded into sterile 96-well plates at a density of 1 × 10^4^ cells per well and allowed to adhere for 24 h. After reaching approximately 70–80% confluence, the culture medium was replaced with fresh medium containing various concentrations (10, 25, 50, 75, and 100 μg/mL) of pure and Nd–ZnO NPs. Control wells received only culture medium without nanoparticles. The cells were incubated for an additional 24 h to allow nanoparticle–cell interaction.

#### MTT assay for cell viability

Following incubation, the nanoparticle-containing medium was carefully aspirated, and the cells were washed twice with phosphate-buffered saline (PBS, pH 7.4) to remove any residual particles. Subsequently, 20 μL of MTT reagent (5 mg/mL in PBS) was added to each well, followed by incubation for 4 h at 37 °C. During this period, viable cells metabolized MTT to insoluble formazan crystals. The supernatant was then discarded, and 150 μL of dimethyl sulfoxide (DMSO) was added to dissolve the formazan. The optical density (OD) of each well was recorded at 570 nm using a microplate reader (Bio-Rad, USA).

Cell viability (%) was calculated using the following equation:

$$Cell\,Viability\,(\% ) = \frac{{OD_{sample} }}{{OD_{control} }} \times 100$$

Each experiment was performed in triplicate, and results were expressed as mean ± standard deviation (SD).

#### AO/EB dual staining for apoptosis and necrosis

To further confirm the mechanism of cell death, AO/EB dual staining was performed. MCF-7 cells were seeded in 6-well plates at a density of 2 × 10^5^ cells per well and treated with IC₅₀ concentrations of ZnO and Nd–ZnO NPs for 24 h. After treatment, the cells were gently washed twice with PBS to remove unbound particles and stained with 1:1 mixture of acridine orange (AO, 100 μg/mL) and ethidium bromide (EB, 100 μg/mL) for 5 min in the dark. Excess dye was removed by washing with PBS, and stained cells were immediately observed under a fluorescence microscope (Olympus IX71, Japan) using a 450–490 nm excitation filter. A minimum of 100 cells per sample were examined in quadruplicate to determine the proportion of viable (green), early apoptotic (green with condensed nuclei), late apoptotic (orange-red fragmented nuclei), and necrotic (red) cells. Morphological features such as nuclear condensation, membrane blebbing, and chromatin fragmentation were documented using digital micrographs.

### Statistical analysis

All statistical analyses were performed using GraphPad Prism 8 software version 8.0.2 (GraphPad Software, Boston, MA, USA). All experiments were conducted in triplicate (n = 3), and results were presented as mean ± SD. Statistical significance between groups was determined using one-way ANOVA followed by Tukey’s post hoc test (*p* < 0.05 was considered statistically significant).

## Result and discussion

### Structural analysis

**Fig. 1:**
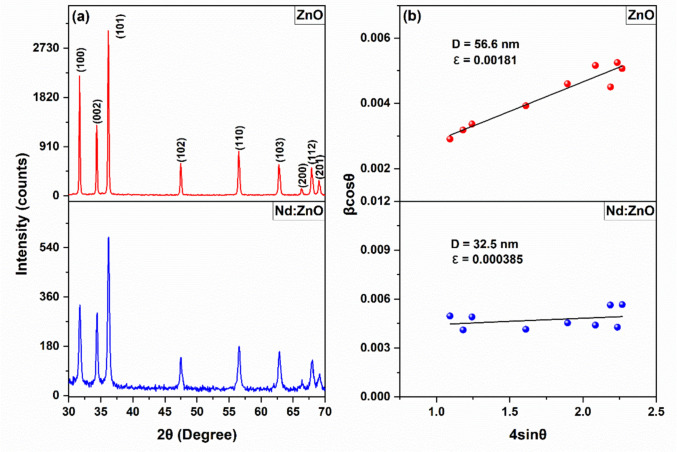
Structural analysis of ZnO and Nd-ZnO NPs. **a** X-ray diffraction (XRD) patterns of pure and neodymium (Nd)-doped ZnO nanoparticles synthesized using *Psidium guajava* leaf extract, confirming the hexagonal wurtzite crystalline structure and showing peak broadening indicative of reduced crystallite size upon Nd incorporation. **b** Williamson–Hall (W–H) plots illustrating the relationship between strain and crystallite size, revealing increased lattice strain and defect formation induced by Nd doping within the ZnO crystal lattice

XRD patterns of pure and Nd-doped ZnO NPs are presented in Fig. 1a, confirming their crystalline nature. The diffraction peaks observed at approximately 31.8°, 34.4°, 36.3°, 47.5°, 56.6°, 62.8°, 66.4°, 67.9°, and 69.1° correspond to the (100), (002), (101), (102), (110), (103), (200), (112), and (201) planes, respectively, characteristic of the hexagonal wurtzite structure (space group P6₃mc), in agreement with the standard JCPDS card no. 36–1451. Notably, no additional peaks attributable to secondary phases or Nd-based oxides were detected, indicating the successful incorporation of Nd^3^⁺ ions into the ZnO lattice without disrupting its fundamental crystal framework. A slight shift of diffraction peaks toward lower 2θ values was observed for Nd-doped ZnO, which can be attributed to the substitution of Zn^2^⁺ ions (ionic radius 0.074 nm) by larger Nd^3^⁺ ions (0.0983 nm), in accordance with Vegard’s law. This substitution induces a marginal increase in lattice parameters; the calculated lattice constants for pure ZnO were *a* = 3.2574 Å and *c* = 5.2158 Å, while Nd-doped ZnO exhibited slightly larger values of *a* = 3.2579 Å and *c* = 5.2153 Å, consistent with the incorporation of Nd^3^⁺ into tetrahedral Zn^2^⁺ sites.

The average crystallite size was estimated using the Williamson–Hall method (Fig. 1b), which accounts for both size broadening and microstrain effects. The results indicate a reduction in crystallite size from 56.6 nm for pure ZnO to 32.5 nm for Nd-doped ZnO. This decrease is attributed to the lattice strain and defect centers introduced by Nd^3^⁺ incorporation, which act as additional nucleation sites and inhibit crystal growth, thereby increasing structural distortion within the ZnO lattice. These findings are consistent with recent studies that have reported similar structural modifications upon Nd doping in ZnO. For instance, a study by (Pascariu et al. 2023) observed a decrease in crystallite size and an increase in lattice strain in Nd-doped ZnO nanostructures, attributed to the incorporation of Nd^3^⁺ ions into the ZnO lattice. Additionally, research by (Kamboj et al. 2023) noted a shift in diffraction peaks and a reduction in crystallite size upon Nd doping, aligning with the observations in the present study.

### Morphology and compositional analysis

**Fig. 2:**
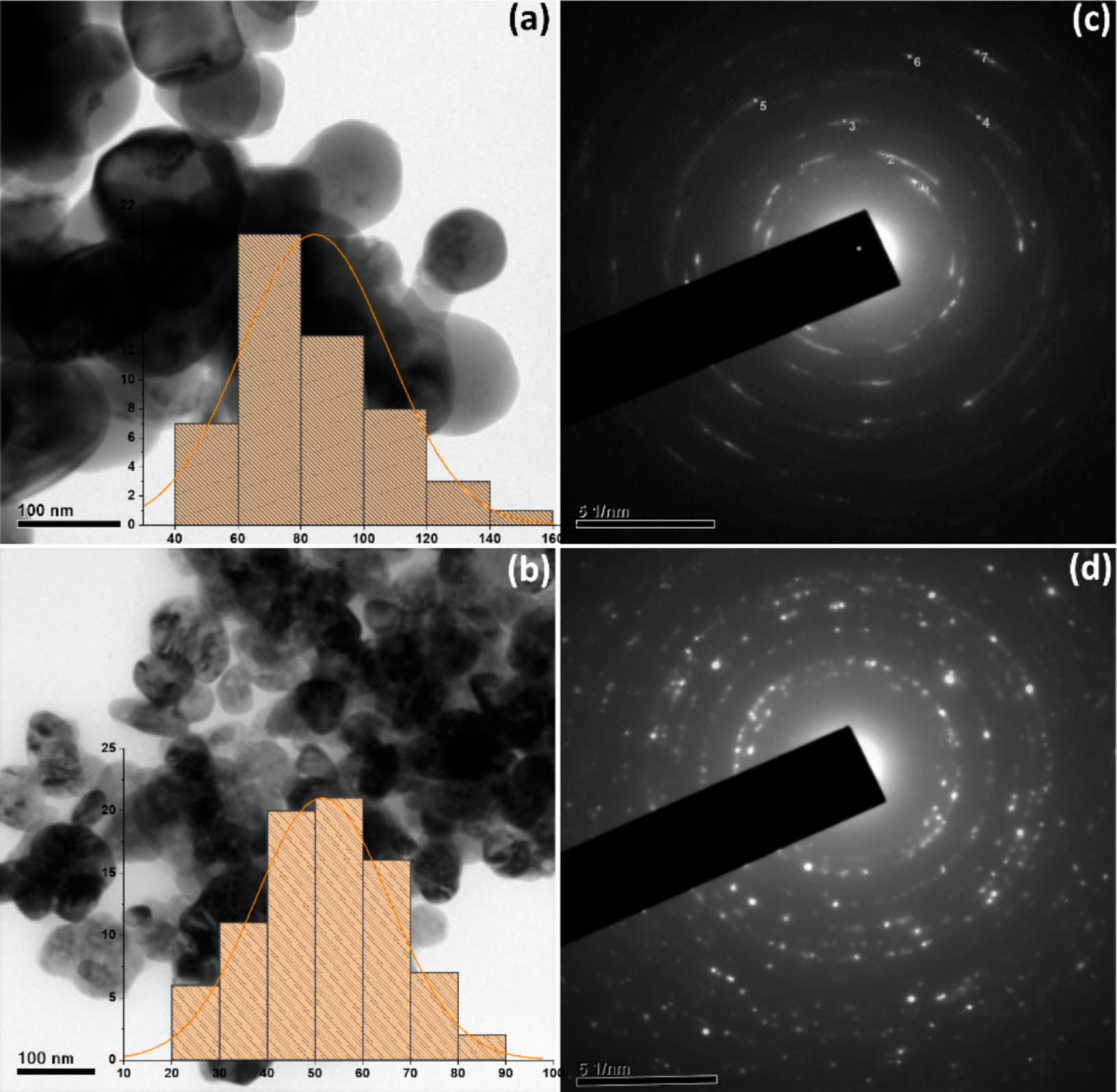
Morphology and compositional analysis of ZnO and Nd-ZnO NPs. **a**, **b** Transmission electron microscopy (TEM) images and **c**, **d** selected area electron diffraction (SAED) patterns of pure and neodymium (Nd)-doped ZnO nanoparticles, respectively. The TEM images reveal well-dispersed, quasi-spherical nanoparticles with reduced particle size upon Nd incorporation, while the SAED patterns confirm their polycrystalline nature consistent with the hexagonal wurtzite structure

TEM analysis was conducted to investigate the morphological characteristics of pure and Nd-ZnO NPs. As depicted in Fig. 2, pure ZnO NPs exhibit relatively larger, well-defined structures, whereas Nd doping leads to noticeable changes in size and dispersion (Fig. 2a). The doped NPs appear smaller and more uniform, suggesting that Nd incorporation affects the growth process, likely due to lattice strain and defect formation (Fig. 2b).

SAED patterns confirm the crystalline nature of both pure and Nd-doped ZnO NPs, displaying distinct diffraction rings corresponding to the hexagonal wurtzite phase of ZnO (Fig. 2c, d). In Nd-doped ZnO, slight broadening of the diffraction rings suggests increased strain and disorder within the crystal lattice, likely due to the incorporation of Nd ions, which have a larger ionic radius than Zn^2^⁺. Particle size distribution analysis further supports these findings, revealing a significant size reduction upon Nd doping. The mean particle size reduces from 80 to 55 nm when Nd is incorporated into ZnO. This reduction can be attributed to Nd’s role in limiting particle growth by introducing additional nucleation sites and preventing the coalescence of growing particles. These results align with the XRD analysis.

**Fig. 3:**
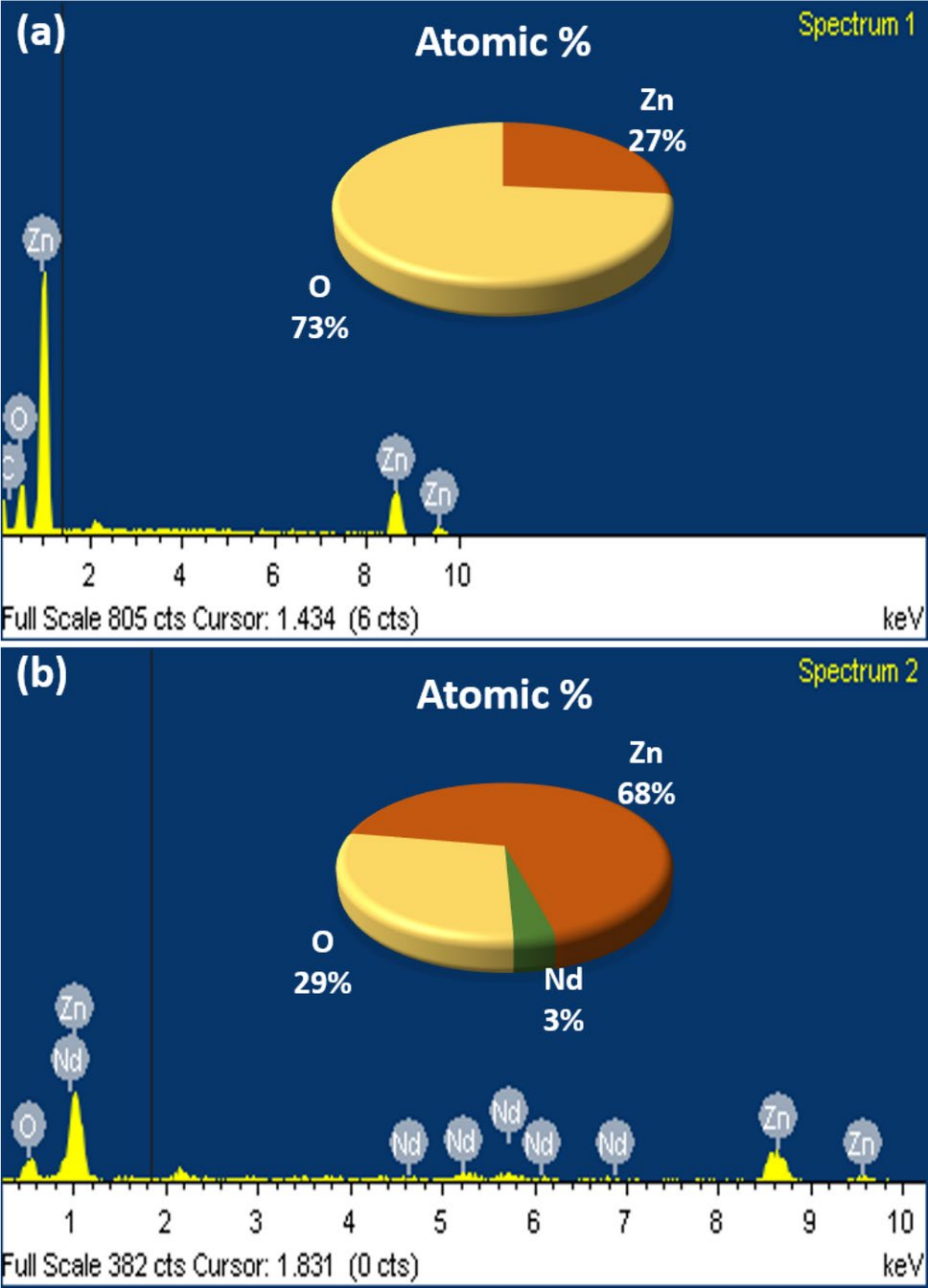
Morphology and compositional analysis of ZnO and Nd-ZnO NPs. Energy-dispersive X-ray (EDX) spectra of **a** pure ZnO and **b** neodymium (Nd)-doped ZnO nanoparticles. The spectra confirm the elemental composition of zinc (Zn) and oxygen (O) in pure ZnO, with the appearance of additional Nd peaks in the doped sample, verifying the successful incorporation of Nd ions into the ZnO lattice without any detectable impurities

EDX spectroscopy confirms the elemental composition and chemical purity of both samples. Peaks corresponding to Zn and O are observed in pure ZnO, while Nd-doped ZnO additionally shows Nd signals, confirming successful doping without detectable impurities (Fig. 3). These findings indicate that Nd incorporation effectively modifies both the structural and morphological characteristics of ZnO nanoparticles, which may enhance their optical, photocatalytic, and biological properties. Recent studies have corroborated these observations. For instance, a study by (Samanta et al. 2019) reported that Nd-doped ZnO nanoparticles exhibited improved dispersion and uniformity, with particle sizes ranging from 22 to 40 nm, aligning with our findings. Additionally, the incorporation of Nd^3^⁺ ions was shown to introduce lattice strain, leading to enhanced photocatalytic activity due to increased surface area and active sites.

### Micro -Raman analysis

**Fig. 4:**
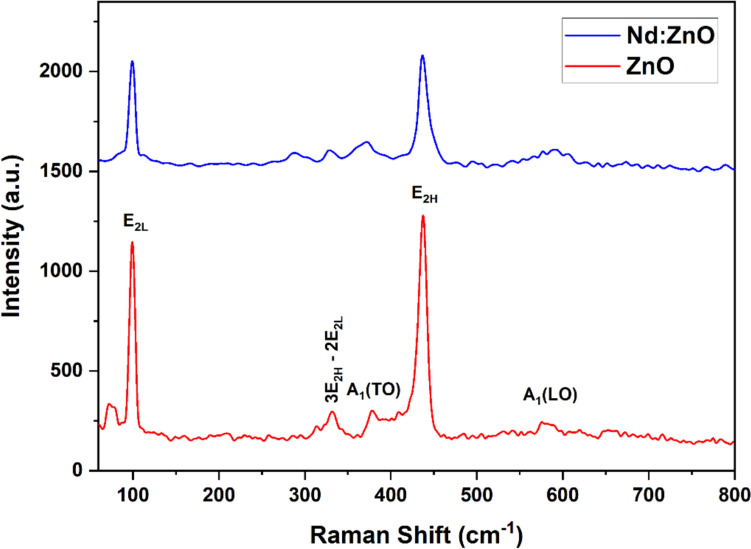
Raman spectra of ZnO and Nd-ZnO NPs. The spectra display characteristic vibrational modes of ZnO, including E₂ (low), E₂ (high), A₁(TO), and A₁(LO), with shifts and intensity changes observed upon Nd doping, indicating lattice distortion, defect formation, and successful incorporation of Nd into the ZnO crystal structure

Raman spectroscopy provides vital insights into lattice dynamics, crystallinity, and defect structures in semiconducting oxides such as ZnO. The Raman spectra of pure and Nd-doped ZnO NPs are shown in Fig. 4, exhibiting characteristic vibrational peaks at approximately 100, 331, 377, 437, and 575 cm⁻^1^. Among these, the well-defined peaks at 100 and 437 cm⁻^1^ correspond to the E₂(low) and E₂(high) modes, respectively, which are characteristic of the wurtzite ZnO lattice. The E₂(low) mode is primarily associated with zinc sublattice vibrations, while the E₂(high) mode corresponds to oxygen vibrations, serving as a fingerprint of high structural order and phase purity in ZnO (Koyano et al. 2002). The sharpness and intensity of these peaks confirm that the synthesized nanoparticles retain a high degree of crystallinity and the hexagonal wurtzite structure.

The relatively weak peak at 331 cm⁻^1^ corresponds to the second-order Raman scattering process arising from the combination of [E₂(high)—E₂(low)] phonons, while the band at 377 cm⁻^1^ represents the A₁(TO) mode. The broad band observed near 575 cm⁻^1^ is assigned to the A₁(LO) phonon mode, which is particularly sensitive to lattice defects, free carrier concentrations, and dopant-induced local strain. In pristine ZnO, this mode is typically linked to intrinsic point defects such as oxygen vacancies (Vₒ), zinc interstitials (Znᵢ), and donor-like free carriers. The enhanced intensity and slight broadening of this peak in Nd-doped ZnO suggest that Nd incorporation modifies the defect environment, promoting the formation of oxygen vacancies and lattice strain rather than passivating these imperfections. Such defect modulation due to rare-earth (RE) doping has been widely reported. For instance, (Pascariu et al. 2023) and (Yang et al. 2018) observed that the incorporation of rare-earth ions such as Nd^3^⁺ and Eu^3^⁺ in ZnO induces local lattice distortion and phonon scattering, leading to a decrease in crystallite size and redshift in Raman-active modes. Similarly, (Kumar 2025) demonstrated that rare-earth-doped ZnO nanostructures exhibit enhanced LO phonon intensity, attributed to the increased concentration of oxygen vacancies and defect-mediated coupling between phonons and free carriers. These modifications are often associated with improved surface reactivity, charge transfer dynamics, and photocatalytic efficiency.

In the present study, the Nd-doped ZnO NPs show a noticeable redshift of Raman peaks toward lower wavenumbers compared to pure ZnO. This shift reflects the lattice expansion and internal strain arising from the substitution of smaller Zn^2^⁺ ions (ionic radius 0.074 nm) with larger Nd^3^⁺ ions (0.0983 nm). The induced tensile strain softens the phonon modes, resulting in lower vibrational frequencies. Furthermore, the decrease in the overall Raman intensity upon Nd doping suggests increased structural disorder or phonon confinement effects, which are consistent with the reduced crystallite size and higher microstrain observed in XRD and TEM analyses. The combined evidence from Raman, XRD, and HRTEM analyses supports the conclusion that Nd doping effectively alters the phonon characteristics and defect structures of ZnO nanoparticles. These changes influence not only the vibrational dynamics but also the electronic and optical properties, enhancing potential applicability in optoelectronic and photocatalytic systems. Comparable studies by (Toma et al. 2022) and (Yatskiv et al. 2023) have shown that rare-earth-doped ZnO nanostructures exhibit modified electron–phonon coupling and improved defect-mediated optical absorption, underscoring the importance of controlled rare-earth incorporation for tailoring ZnO’s multifunctional properties.

### XPS study

**Fig. 5:**
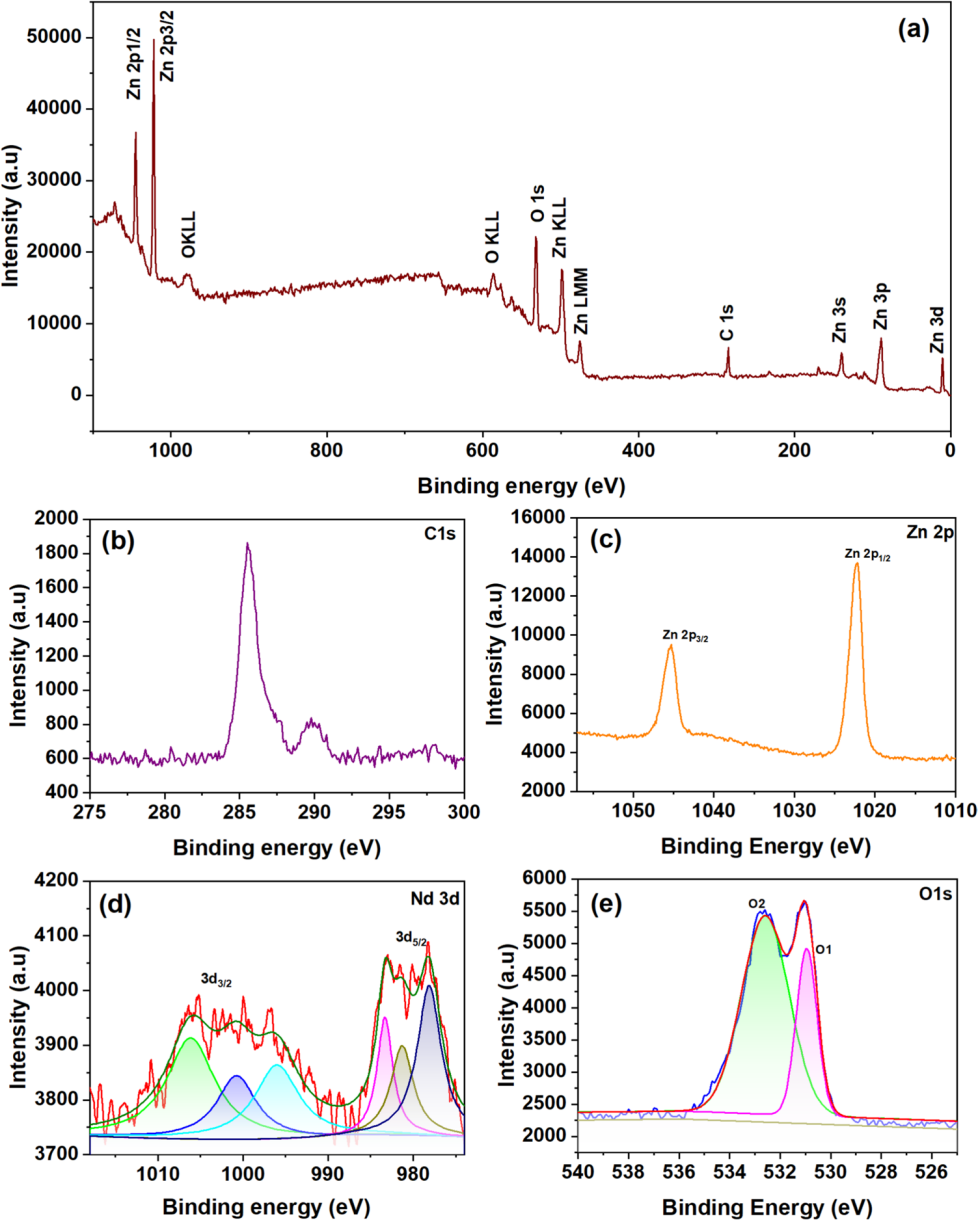
X-ray photoelectron spectroscopy (XPS) analysis of ZnO and Nd-ZnO NPs. **a** survey spectrum confirming the presence of Zn, O, and Nd; high-resolution core-level spectra of **b** C 1 s, **c** Zn 2p, **d** Nd 3d, and **e** O 1 s, illustrating elemental composition, oxidation states, and successful Nd incorporation into the ZnO lattice

XPS was employed to investigate the surface chemical composition and oxidation states of the elements in the synthesized ZnO and Nd-doped ZnO NPs. The survey spectrum (Fig. 5a) confirmed the presence of Zn, O, and Nd, verifying the successful incorporation of neodymium into the ZnO lattice. The appearance of characteristic Nd peaks without any additional impurity signals indicates that Nd was effectively substituted into the ZnO matrix rather than forming separate Nd-based oxide phases. Similar elemental profiles were observed in recent studies on rare-earth-doped ZnO nanostructures, such as Ce-, La-, and Gd-doped ZnO, where homogeneous dopant distribution was achieved without altering the wurtzite phase (Dhiman et al. 2023).

The high-resolution Zn 2p spectrum (Fig. 5c) exhibited two distinct peaks at binding energies of 1022.2 eV and 1045.4 eV, corresponding to Zn 2p₃/₂ and Zn 2p₁/₂, respectively. The spin–orbit separation of 23.2 eV agrees with standard values for Zn^2^⁺ in ZnO, confirming that Zn maintained its + 2 oxidation state within the hexagonal wurtzite structure (Ghoshal et al. 2008). No significant shifts in Zn 2p binding energies were observed, suggesting that Nd incorporation caused minimal lattice distortion around Zn sites, consistent with prior reports that moderate rare-earth doping (below 5 mol%) does not alter Zn^2^⁺’s local electronic environment (Pascariu et al. 2023).

The Nd 3d spectrum (Fig. 5d) revealed two well-defined peaks at 977.9 eV (Nd 3d₅/₂) and 1006.2 eV (Nd 3d₃/₂), characteristic of Nd^3^⁺ species. The presence of weak satellite features attributed to shake-up transitions further supports the trivalent oxidation state of neodymium and its successful substitution for Zn^2^⁺ in the lattice. This finding is consistent with XPS results from other Nd-doped metal oxides, such as Nd–TiO₂ and Nd–SnO₂, where the Nd^3^⁺ state enhances electron density near the conduction band, leading to improved charge separation and photocatalytic performance (Trujillo-Navarrete et al. 2017). In ZnO, Nd^3^⁺ doping introduces localized electronic states below the conduction band, which can reduce the bandgap energy and facilitate visible-light absorption, an effect corroborated by optical and PL studies (Lal et al. 2023).

The O 1 s spectrum (Fig. 5e) exhibited two main peaks centered at 531.0 eV and 532.8 eV. The lower-energy peak (531.0 eV) is attributed to lattice oxygen (O^2^⁻) bonded to Zn^2^⁺, whereas the higher-energy component (532.8 eV) corresponds to oxygen in Nd–O environments and surface-adsorbed oxygen species. The relative intensity increase of the 532.8 eV peak in the doped sample implies that Nd incorporation induces surface oxygen vacancies or chemisorbed oxygen, which are known to enhance redox reactivity and catalytic activity. Comparable oxygen-state modifications have been reported in Nd-doped ZnO thin films, where Nd^3^⁺ ions generate charge imbalance compensated by oxygen vacancies, improving carrier mobility and photocatalytic behavior (Sahu et al. 2021).

Taken together, the XPS results substantiate that Nd^3^⁺ ions successfully substitute Zn^2^⁺ in the ZnO lattice while maintaining the wurtzite crystal framework. The coexistence of lattice oxygen and Nd–O bonds, accompanied by the emergence of oxygen vacancies, indicates a balanced defect structure that can significantly influence the electronic and optical properties of Nd-doped ZnO nanoparticles. This is in strong agreement with recent works highlighting that controlled rare-earth doping enhances photocatalytic, antibacterial, and optoelectronic performance by tailoring surface chemistry and defect states (Vindhya et al. 2023; Li et al. 2023; 2025).

### Photoluminescence study

**Fig. 6:**
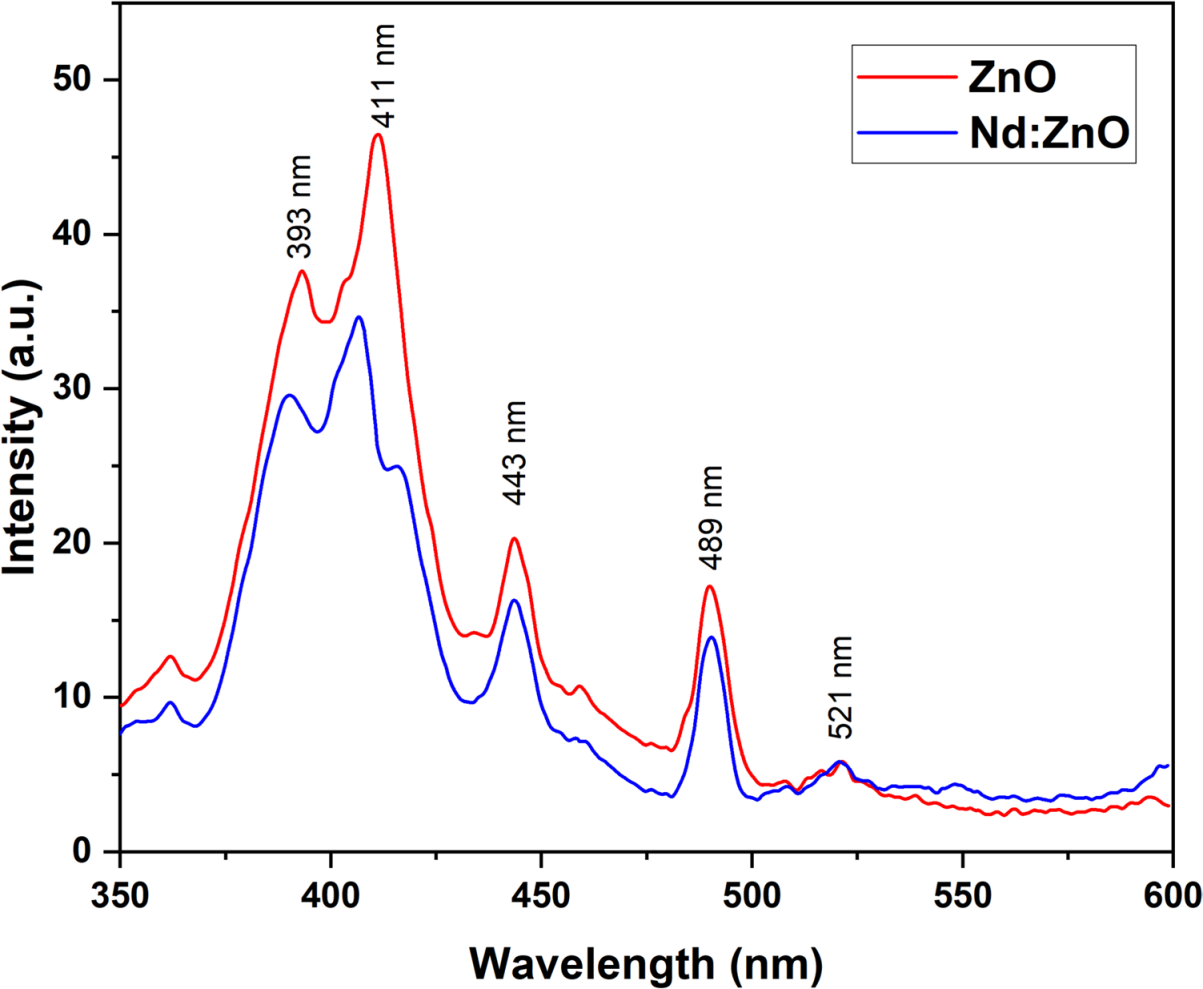
Photoluminescence (PL) spectra of ZnO and Nd-ZnO NPs, showing the emission profiles and defect-related luminescence changes induced by Nd incorporation

Figure 6 presents PL spectra of the synthesized ZnO NPs in both pure and Nd-doped forms under an excitation wavelength of 320 nm. Both spectra exhibit broad visible emission ranging from 350 to 650 nm, revealing the coexistence of near-band-edge and deep-level emissions arising from various defect centers and excitonic transitions within the ZnO lattice. The PL profile displays five prominent emission bands centered at approximately 393, 410, 443, 489, and 521 nm, corresponding to distinct electronic transitions associated with intrinsic and extrinsic defects. The 393 nm peak represents the NBE emission, originating from the radiative recombination of free excitons near the band edge of ZnO. This emission is a hallmark of the material’s crystalline integrity and optical quality. The violet emission at around 410 nm corresponds to interstitial zinc (Znᵢ) defects, which introduce shallow donor levels slightly below the conduction band, enabling radiative sub-bandgap transitions (Fan et al. 2005). The blue emission observed at 443 nm is attributed to singly ionized oxygen vacancies (Vₒ⁺), one of the most dominant intrinsic defects in ZnO. These vacancies serve as deep-level donors, where electrons recombine with holes in the valence band or acceptor states. The green emission near 489 nm arises from oxygen interstitials (Oᵢ), while the yellow emission around 521 nm is linked to deeply trapped electrons recombining with photogenerated holes, likely associated with oxygen-rich environments or surface-adsorbed oxygen species (Lim et al. 2021).

Upon Nd doping, a noticeable decrease in overall PL intensity is observed, accompanied by a slight redshift in emission peaks. This attenuation in luminescence intensity suggests that Nd^3^⁺ ions introduce additional non-radiative recombination centers within the ZnO lattice, effectively quenching the radiative processes. Nd^3^⁺ ions, possessing unfilled 4f orbitals, can act as electron traps, facilitating charge carrier recombination through non-radiative pathways (Reddy 2025). This mechanism is consistent with findings by (Pascariu et al. 2023), who reported reduced PL intensity in Nd-doped ZnO nanostructures due to increased structural disorder and carrier trapping. Similarly, (Xian and Li 2013) observed a redshift and quenching of NBE emission in Nd-doped ZnO films, attributed to energy transfer between ZnO excitons and Nd^3^⁺ f–f transitions. The observed redshift in the PL spectra may also be explained by lattice strain and defect-induced band tailing effects introduced by Nd incorporation. As Nd^3^⁺ ions (0.0983 nm) replace Zn^2^⁺ ions (0.074 nm), lattice distortion occurs, altering the electronic band structure and reducing the energy of emitted photons (Ashokkumar et al. 2023). Furthermore, the enhancement of defect-related DLE emissions indicates that Nd doping modifies the defect equilibrium by promoting oxygen vacancy formation or stabilizing zinc interstitials. This behavior aligns with reports by (Layek et al. 2016) and (Jayswal and Moirangthem 2024), who were demonstrated that rare-earth dopants induce lattice strain and defect complex formation, effectively tailoring optical emission characteristics.

In addition, Nd^3^⁺ ions are known to introduce localized 4f energy levels within the ZnO bandgap, facilitating possible energy transfer processes between host excitons and dopant ions. This mechanism has been proposed to enhance visible-light absorption and extend carrier lifetimes, beneficial for photocatalytic and optoelectronic applications (Zhao et al. 2014). Studies by (Toma et al. 2022) and (Susarrey-Arce et al. 2009) also reported similar quenching phenomena and emission modulation in rare-earth-doped ZnO systems, where dopant-induced trap states significantly affected recombination kinetics and luminescence yield. Overall, the PL analysis confirms that Nd doping effectively tunes the optical response of ZnO nanoparticles by introducing structural distortions, modifying defect states, and establishing non-radiative recombination channels. These alterations enhance the potential of Nd-doped ZnO for applications in visible-light-driven photocatalysis, photonic devices, and bioimaging, where defect engineering and dopant-induced band modification play crucial roles.

### Photocatalytic activity

**Fig. 7:**
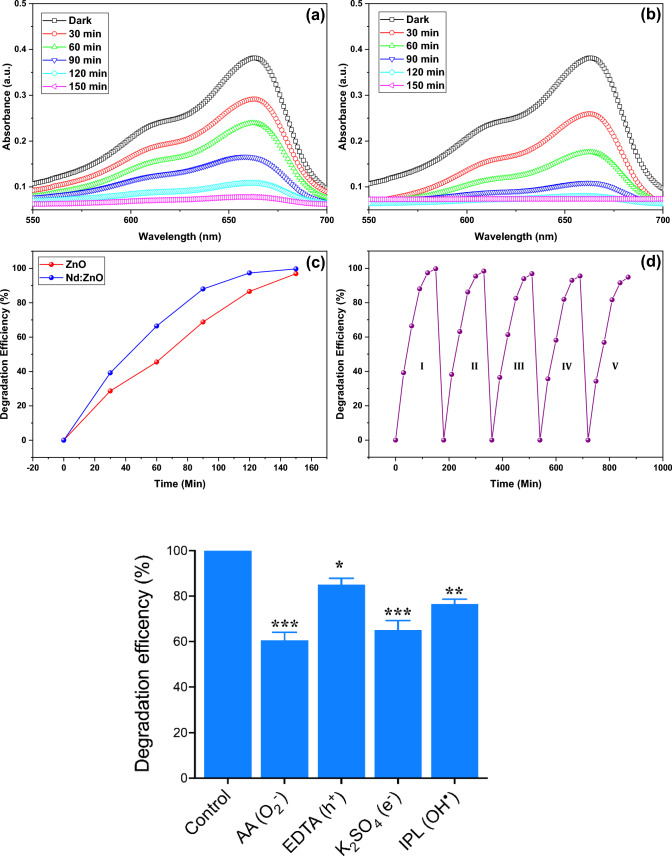
Photocatalytic degradation of methylene blue (MB) under sunlight irradiation. **a** UV–Vis absorbance spectra of MB in the presence of pure ZnO nanoparticles; **b** UV–Vis absorbance spectra of MB with Nd-doped ZnO nanoparticles; **c** Comparative degradation efficiency of ZnO and Nd:ZnO nanoparticles; **d** Reusability and stability of Nd:ZnO photocatalyst over five consecutive cycles

The photocatalytic degradation of MB by pure ZnO and Nd-doped ZnO NPs was systematically examined under natural sunlight irradiation for 150 min. As depicted in Fig. 7a,b, the absorption peak intensity of MB steadily decreased throughout the irradiation period, with the solution becoming almost colorless at the end of the reaction. Both catalysts demonstrated notable degradation capabilities; however, Nd-doped ZnO NPs exhibited a markedly enhanced photocatalytic performance, achieving approximately 99% degradation efficiency (Fig. 7c). The superior photocatalytic efficiency of Nd-doped ZnO NPs can be primarily attributed to the effective generation of ROS via redox interactions between photogenerated electrons and holes. Upon sunlight exposure, electrons in the conduction band and holes in the valence band initiate redox reactions that produce hydrogen peroxide (H₂O₂), hydroxyl radicals (·OH), and superoxide anions (O₂·⁻). Similar mechanisms have been reported in doped ZnO systems, where rare-earth elements enhance ROS yield by improving charge carrier dynamics (Pratomo et al. 2025).

The recyclability and stability of Nd-ZnO NPs were assessed over five consecutive photocatalytic cycles (Fig. 7d). The degradation efficiency remained consistently high, with only a slight decline attributed to material loss during handling rather than structural deterioration. XRD and FTIR analyses (not shown) confirmed that the crystalline structure and functional groups of the catalyst remained intact after repeated use. These findings underscore the robust stability and reusability of Nd-ZnO NPs, which is consistent with previous reports demonstrating long-term photocatalytic durability in rare-earth-doped ZnO systems (Dhiman et al. 2023). Our previous scavenger-based study further confirmed that O₂·⁻ and photogenerated electrons play dominant roles in MB degradation (Ashokkumar et al. 2023). This observation aligns with reports that doping ZnO with lanthanides (e.g., Nd3⁺, Ce3⁺, and La3⁺) introduces localized states within the bandgap, facilitating electron trapping and delaying recombination (Irtiqa and Rahman 2020; Kumar and Kavitha 2021). PL analyses revealed that Nd doping effectively suppresses radiative recombination of charge carriers, resulting in prolonged lifetimes of photogenerated charges and enhanced ROS production. Additionally, the Nd-ZnO NPs exhibited smaller crystallite sizes and larger surface areas than pure ZnO, as corroborated by TEM analysis. Such morphological refinements are known to increase the number of surface-active sites, thereby promoting adsorption and subsequent photodegradation of MB molecules (Kumar and Sahare 2012). Collectively, these structural and electronic modifications underlie the superior photocatalytic activity of Nd- ZnO NPs.

To elucidate the kinetics of MB degradation, both pseudo-zero-order and pseudo-first-order models were employed. The corresponding rate constants, regression coefficients (R2), and half-lives (t₁/₂) are summarized in Table 1. Under the zero-order model, Nd-doped ZnO exhibited a higher rate constant (k₀ = 6.61 × 10⁻^3^ mg·L^−1^·min^−1^) and a shorter half-life (75 min) compared to pure ZnO (k₀ = 6.39 × 10⁻^3^ mg·L^−1^·min^−1^; t₁/₂ = 78 min). In zero-order kinetics, the reaction predominantly occurs at surface-active sites. Hence, the enhanced k₀ of Nd-ZnO NPs indicates an increased density and reactivity of surface sites due to Nd incorporation (Tarasenka et al. 2022). The first-order kinetic model further substantiated these findings, with the rate constant (k₁) increasing from 21.8 × 10⁻3 min⁻1 (pure ZnO NPs) to 36.8 × 10⁻3 min⁻1 (Nd- ZnO NPs). The corresponding half-life decreased from 32 to 19 min, confirming that Nd doping significantly accelerates degradation kinetics through improved charge separation and ROS production. Similar enhancements in first-order kinetic behavior have been reported for Sm-, Gd-, and Ce-doped ZnO systems, where rare-earth dopants promote efficient interfacial charge transfer and extend visible-light response (Sood et al. 2025). The R2 values (0.9831 for zero-order in pure ZnO vs. 0.9325 for first-order in Nd-ZnO NPs) suggest that Nd doping shifts the reaction mechanism toward a first-order pathway. This shift implies a greater dependence on MB concentration, consistent with improved photon utilization and more dynamic surface–dye interactions facilitated by Nd-induced charge redistribution.

**Table 1:** Kinetics parameters for degradation of MB dye in the presence of the pure and Nd doped ZnO nanoparticles

Samples	Zero order Model			First order Model		
	K_0_ × 10^–3^ (mg/Lmin)	*R*^2^	t_1/2_ (min)	K_1_ × 10^–3^ (min^−1^)	*R*^2^	t_1/2_ (min)
ZnO	6.39	0.9831	78	21.8	0.9016	32
Nd:ZnO	6.61	0.8978	75	36.8	0.9325	19

The role of individual ROS species in the degradation process was investigated through radical scavenging experiments (Fig. 7e). Ascorbic acid (AA), ethylenediaminetetraacetic acid (EDTA), potassium sulfate (K₂SO₄), and isopropanol (IPA) were used to quench O₂·⁻, h⁺, e⁻, and ·OH radicals, respectively. The degradation efficiencies after 150 min of sunlight irradiation in the presence of these scavengers were 59%, 83%, 62%, and 79%, respectively. These results indicate that photogenerated electrons and superoxide radicals are the principal oxidative species, whereas hydroxyl radicals and valence band holes play secondary roles. Similar mechanistic trends have been observed in other doped ZnO systems, where O₂·⁻ was identified as the dominant ROS driving dye degradation (Halim et al. 2025). Nd3⁺ ions serve as electron traps that suppress recombination, facilitating efficient electron transfer to adsorbed O₂ molecules and consequently generating higher concentrations of superoxide radicals (Liu et al. 2021). The enhanced ROS generation directly correlates with the observed kinetic acceleration and degradation efficiency.

The present findings confirm that Nd doping significantly enhances ZnO’s photocatalytic activity through synergistic improvements in charge separation, surface reactivity, and ROS generation. The dual kinetic behaviour combining accelerated degradation and altered reaction order suggests that Nd not only improves the intrinsic catalytic performance but also modifies the reaction mechanism itself. Such doped systems offer great potential for solar-driven environmental remediation, particularly in the degradation of persistent organic pollutants in wastewater. Comparable improvements in photocatalytic activity have been documented in Ce-doped TiO₂ (Li et al. 2024) and Sm-doped ZnO (Jerlin Jose et al. 2017), further emphasizing the role of rare-earth elements in extending visible-light absorption and stabilizing photogenerated charge carriers. Therefore, Nd-ZnO NPs emerges as a promising photocatalyst with excellent efficiency, stability, and practical viability for green environmental applications.

### Anticancer Potentials of Green Synthesized ZnO/Nd-ZnO NPs

**Fig. 8:**
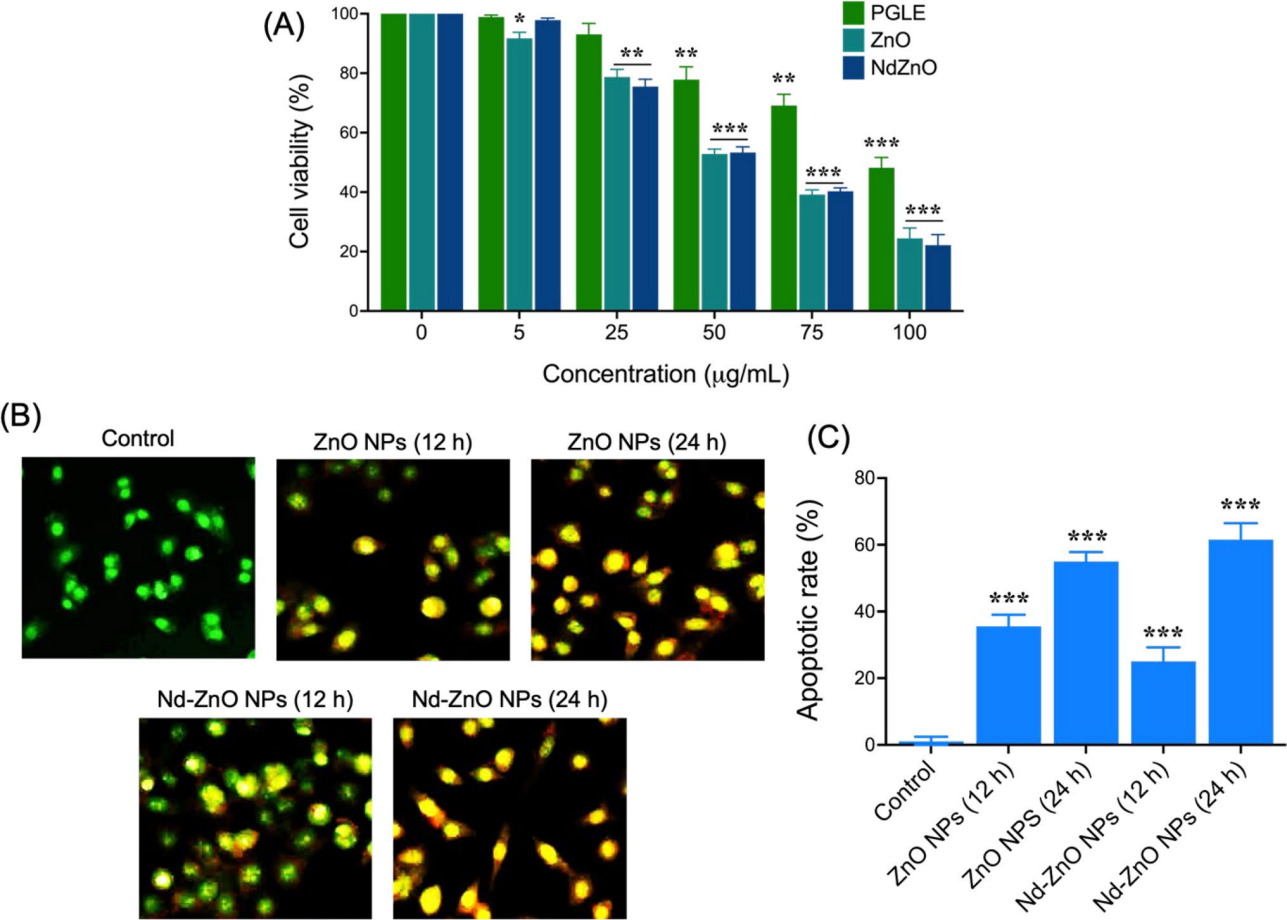
Anticancer activity of PGLE, ZnO NPs, and Nd-ZnO NPs against MCF-7 breast cancer cells. **a** MTT assay showing cell viability after treatment with PGLE, ZnO NPs, and Nd-ZnO NPs; **b** Morphological analysis using AO/EB staining. Control cells exhibit uniform green fluorescence, whereas treatment with ZnO and Nd-ZnO NPs (100 µg/mL) for 12 h and 24 h shows increased orange-red apoptotic cells; **c** Histogram representing the percentage of apoptotic cells. Data are expressed as mean ± SD of three independent experiments. Statistical significance: *p < 0.05, **p < 0.01, ***p < 0.001 compared to control

The cytotoxicity of PGLE, ZnO NPs, and Nd–ZnO NPs toward human breast adenocarcinoma (MCF-7) cells was evaluated using the MTT assay. As shown in Fig. 8a, both nanoparticle types significantly reduced cell viability in a concentration-dependent manner. At lower concentrations (10–25 µg/mL), cytotoxicity was minimal; however, at higher concentrations, a sharp decline in viability was observed. At 100 µg/mL, the viability decreased to approximately 35% for pure ZnO and to 25% for Nd–ZnO NPs. The lower IC₅₀ value of Nd–ZnO NPs indicates that Nd doping enhances cytotoxic potential by modifying the crystal structure, surface charge, and electronic properties, thereby promoting stronger particle–cell interactions and oxidative stress (Wu et al. 2023). Indeed, PGLE alone induced a significant and dose-dependent reduction in cell viability, consistent with previous reports (Lok et al. 2023). However, the decrease in cell viability was markedly greater in cells treated with PGLE-mediated green-synthesized ZnO NPs and Nd-ZnO NPs. These findings demonstrate that the green-synthesized Nd-ZnO NPs exhibited enhanced cytotoxic activity against human breast cancer cells in vitro.

The mechanism of cell death was further investigated using acridine AO/EB dual staining. Fluorescence microscopy (Fig. 8b) showed that control cells displayed uniform green fluorescence, characteristic of viable cells with intact membranes. In contrast, ZnO Nps-treated cells exhibited mixed green and orange fluorescence, indicating early apoptosis, while Nd–ZnO NPs-treated cells showed intense orange-red fluorescence with nuclear condensation and fragmentation, consistent with late apoptotic and necrotic stages. Quantitative analysis (Fig. 8c) revealed a higher percentage of apoptotic cells in the Nd–ZnO NPs group compared to the pure ZnO NPs group, confirming enhanced apoptosis induction by neodymium doping, which is highly consistent with previous observations (Mathivanan and Kumar 2019). The increased apoptotic activity of Nd–ZnO NPs can be primarily attributed to the elevated generation of ROS within the cells. Doping with rare-earth ions improves electron–hole pair separation and facilitates greater ROS production under physiological conditions (An et al. 2024). Elevated ROS levels disrupt mitochondrial membranes, trigger cytochrome c release, and activate caspase-dependent apoptotic pathways (Ricci et al. 2003). The observed morphological features, including cell shrinkage, membrane blebbing, and chromatin condensation, further confirm that apoptosis is the primary mechanism of cell death. These findings are consistent with previous studies reporting that rare-earth-doped ZnO nanoparticles exhibit enhanced cytotoxic and apoptotic effects compared with undoped ZnO due to increased oxidative stress and DNA fragmentation (Al Bitar et al. 2023; Theivarasu and Indumathi 2017; Hannachi et al. 2022). Moreover, doped ZnO nanoparticles often show selective toxicity toward cancer cells while sparing normal cells, which is advantageous for biomedical applications (Ibraheem et al. 2022; Li et al. 2010). These findings highlight the potential of neodymium doping as a strategy to fine-tune the physicochemical and biological properties of ZnO nanomaterials for targeted cancer therapy. Although AO/EB dual staining in the present study provided clear morphological evidence of apoptosis induction by Nd-ZnO NPs in MCF-7 cells, we acknowledge that molecular-level validation of apoptosis-related markers (e.g., caspases, Bax, and Bcl-2) was not performed. Future studies will focus on detailed protein and gene expression analyses, along with in vitro and in vivo models, to further elucidate the underlying molecular mechanisms of ZnNd NP-induced apoptosis.

## Conclusion

In this study, pure and Nd-doped ZnO NPs were successfully synthesized using *P. guajava* leaf extract through an eco-friendly green synthesis approach. Structural and morphological analyses confirmed the formation of hexagonal wurtzite ZnO with reduced particle size and increased defect density upon Nd incorporation. Spectroscopic studies revealed that Nd doping significantly influenced the optical and photoluminescent properties by introducing defect states and enhancing visible-light absorption. The photocatalytic experiments demonstrated superior degradation efficiency of methylene blue under sunlight for Nd–ZnO compared to pure ZnO, attributed to improved charge separation and higher ROS generation. Furthermore, cytotoxicity and fluorescence assays against MCF-7 human breast cancer cells indicated potent anticancer activity, with apoptosis identified as the primary mechanism of cell death. These findings suggest that Nd doping not only enhances the photocatalytic and optical performance of ZnO NPs but also augments their biomedical efficacy. Overall, the use of *P. guajava* extract provides a sustainable, biocompatible, and efficient route for producing multifunctional ZnO-based nanomaterials with potential applications in environmental remediation and cancer therapy, bridging the gap between green nanotechnology and rare-earth material innovation.

## Data Availability

The datasets generated during and/or analysed during the current study are available from the corresponding author on reasonable request.
